# Notice

**Published:** 1860-03

**Authors:** 


### NOTICE.

Having repaired the loss of twenty-four hours ago, by a new programme of subjects, we trust that it will cost no one any thing, except a hard day's work for ourselves. If the finder of our manuscript for March can make any thing out of it, or can decipher enough to learn that it belongs to 42 Irving Place, we may be saved some work for April. If it never comes to hand, we will only say, that there were valuable crumbs of comfort given to Northern men with Southern principles, or Southern principles with Northern men, or Northern principles Southern, or something of that sort. It's all mixed up some how, and our mishap has put us in such a hurry we hadn't half time to enjoy the turkey-dinner to-day specially provided for our children, who were all at home from school. Our Southern correspondent who inquires of us so courteously to know whether we were on the fence, on the great black and white question, has, no doubt, leisure to unravel the complication. Most Southerners have time, because they have other people to do the work for them. Wish some body would come and work for us a spell. In the event, however, that he is not disposed to thus employ himself, we advise that he purchase the six bound volumes of Hall's Journal of Health, price only seven dollars! and he will be certain to find interspersed through its pages, not only that we have thought and written about slavery, but that much good advice about health and good morals has also been given, the practice of which will be well calculated to make him a wise, healthy, happy, and good man, if he is not so already. We will vouchsafe this much, however, that in a sense we are on the fence, which pre-supposes that we are also out of the mud, above the fierce combatants; looking complacently down on the indignation of the one, and the needless excitement of the other. It must not be supposed, however, that we have no creed, or are afraid of our faith! Not a whit more than the editors of the *New-York Observer* are supposed by some to be. Our principles are solid, substantial, valuable. They are the principles of nine-tenths of the sensible and insensible men North and South, with the women and children thrown in. In fact, we go with the majority. Like the London *Times* we sail with the tide. We follow the straws. That is the reason we get along so smoothly. Having thus plainly declared our creed, we take leave, with all due respect, of our Southern correspondent, with the request that he will send us a telegram, at his earliest convenience, when he has got hold of the right end of the string.

N. B.—To our other subscribers, who have not the advantage of having the "key" which is in the possession of our correspondent, we will merely say that the principles of which we have spoken in such high terms are seven in number, to wit: "The five loaves and two fishes."

